# Mold Flow Analysis of Motor Core Gluing with Viscous Flow Channels and Dipping Module

**DOI:** 10.3390/polym13132186

**Published:** 2021-06-30

**Authors:** Yong-Jie Zeng, Sheng-Jye Hwang, Yu-Da Liu, Chien-Sheng Huang

**Affiliations:** 1 Department of Mechanical Engineering, National Cheng Kung University, Tainan 701, Taiwan; tyc861220@gmail.com; 2 Metal Industry Research and Development Center, Kaohsiung 811, Taiwan; mouda420@gmail.com (Y.-D.L.); ccjames.huang@gmail.com (C.-S.H.)

**Keywords:** motor core, iron sheet, computer-aided engineering tools, gluing

## Abstract

A motor core is formed by stacking iron sheets on top of each other. Traditionally, there are two stacking methods, riveting and welding, but these two methods will increase iron loss and reduce usage efficiency. The use of resin is the current developmental trend in the technology used to join iron sheets, which has advantages including lowering iron loss, smoothing magnetic circuits, and generating higher rigidity. The flow behavior of resin in gluing technology is very important because it affects the dipping of iron sheets and the stacking of iron sheets with resin. In this study, a set of analytical processes is proposed to predict the flow behavior of resin through the use of computer-aided engineering (CAE) tools. The research results are compared with the experimental results to verify the accuracy of the CAE tools in predicting resin flow. CAE tools can be used to predict results, modify modules for possible defects, and reduce the time and costs associated with experiments. The obtained simulation results showed that the filling trend was the same as that for the experimental results, where the error between the simulation results for the final dipping process and the target value was 0.6%. In addition, the position of air traps is also simulated in the dipping process.

## 1. Introduction

In the motor core manufacturing process, one of the sources of performance degradation is the joining method used during the stacking of iron sheets. From Krings’ [1] research, it can be seen that in addition to the joining method in the stacking process, the cutting method and the various combinations of iron sheets will have an impact on iron loss. Lamprecht’s [2,3] research pointed out that in the iron sheet stacking process, the use of interlocking or welding methods will increase iron loss. The results were verified both experimentally and using the finite element method. Therefore, that research discusses the use of gluing technology to reduce iron loss and improve the efficiency of this process. The resin flow in gluing technology is very important. Due to the design of the viscous flow channel and the dipping module, the resin flows into an open space and forms a free surface in contact with the air, after which the iron sheet is dipped into resin. This is similar to the relationship between a stamp (iron sheet) and an ink pad (free surface). Finally, the iron sheets with resin are stacked on each other to form a motor core. In the present study, mold flow analysis software is used to predict the resin flow and analyze the iron sheet dipping process. Chang [4] established a three-dimensional numerical model to simulate the filling behavior of EMC (epoxy molding compound) in IC packaging materials. Based on the consistency between the experimental and simulation results, the accuracy of this method in the filling process of a three-dimensional model was verified. In a mold flow analysis, the free surface produced by the contact between the fluid and the air cannot be ignored. Aniszewski [5], Srinivasan [6], and Zhou [7] studied using the fluid volume method to solve the free surface problem, and the simulations were verified to be predictive. Khalil Abdullah’s [8] and Chen’s [9] research pointed out the Cross Castro–Macosko model is stable and reliable on the flow rheology. Azmi’s [10] and Duan’s [11] research show that using the the Cross Castro–Macosko model has good results in predicting the flow behavior of thermoset materials. In Chen’s [9] and Domínguez’s [12] research, it can be seen that Kamal’s model is a good approach to predict the change in the mechanical degree of conversion during the material curing process. Lai’s [13] research shows the 3D mold flow modeling capabilities of Moldex3D, and the use of the Cross Castro–Macosko model and Kamal’s model to obtain accurate results for thermoset materials.

In the present research, a discussion is made of the integration of gluing technology in the stamping process. A set of analytical methods is proposed to predict the flow behavior of resin, the results of which are used in the dipping analysis. In the case of resin flow, the fluid volume method was used to calculate the free surface that occurs when the fluid is in contact with the air.

## 2. Experiment

### 2.1. Equipment

**Figure 1:**
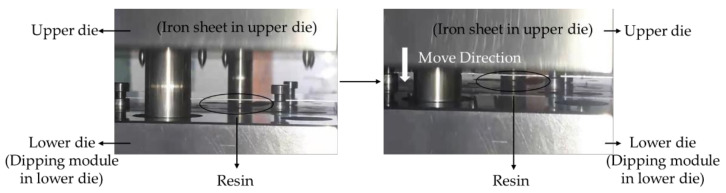
Motor core manufacturing process steps.

The resin flows between the upper and lower die due to the design of the runner and the dipping module, and the iron sheet that moves with the upper die for dipping can be seen in Figure 1. In the experiment, the resin is pushed by a pump at a pressure of 0.8 MPa; the stamping equipment is set to 50 strokes per minute (50 SPM); the stamping force is 0.022 tf, and the stamping speed is 1.83 mm/s. The stamping equipment was produced by the Metal Industries Research and Development Center, Kaohsiung, Taiwan.

**Figure 2:**
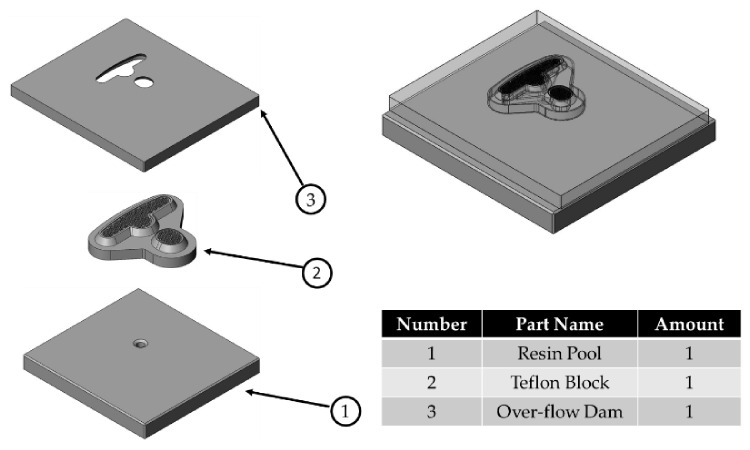
Runner design.

**Figure 3:**
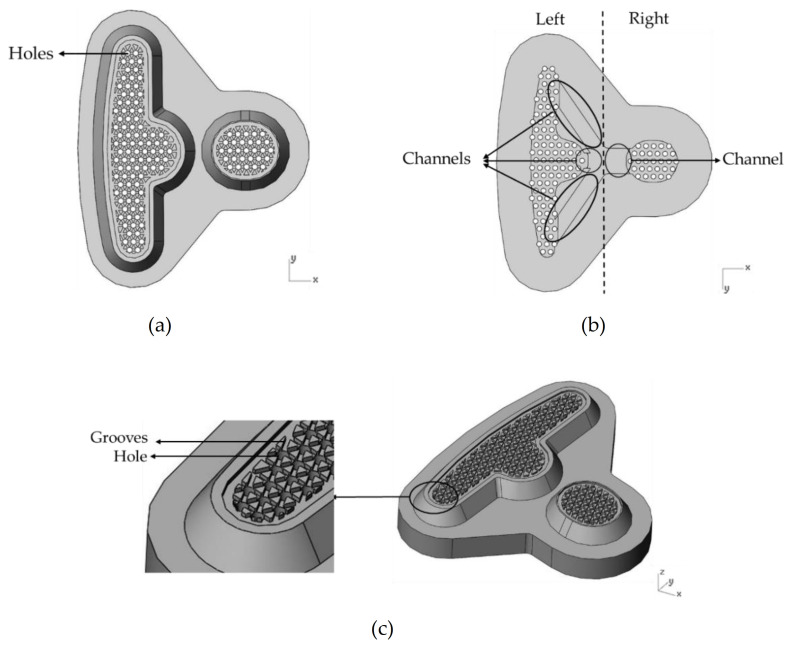
The design of the Teflon module. (**a**) Top view of the Teflon block. (**b**) Bottom view of the Teflon block. (**c**) The microstructure of the Teflon block.

The runner design comprises three parts: the resin pool, the over-flow dam, and a Teflon block (see Figure 2). In addition, Figure 3 shows the design of the Teflon module. In Figure 3a, there are many holes in the top view. It is a Teflon microstructure with a 1 mm diameter and 1.5 mm spacing. Figure 3b is a bottom view of the Teflon block revealing the internal design of the Teflon block. The resin flowing into the resin pool is divided into the left and right sides. The left half has three flow channels, while the right half has one. Figure 3c shows the grooves next to the hole, which are designed to make the resin flow evenly. The purpose of this study is to explore the influence of the design of the runner and the module on the resin flow.

### 2.2. Material

**Figure 4:**
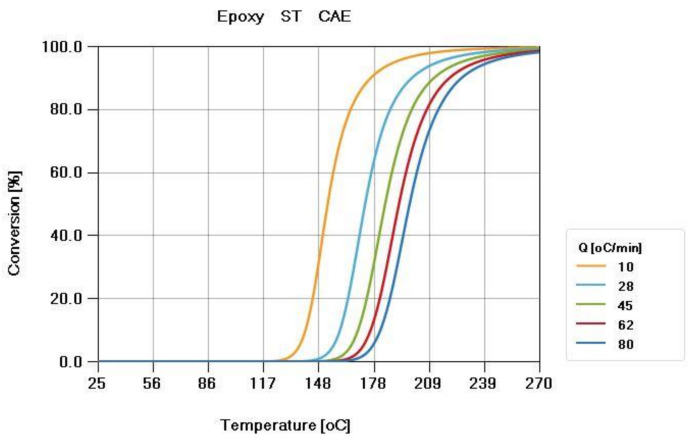
Curing kinetics curve of the ST resin. The curing kinetics is measured by a DSC with different temperature ramping rates (Q = 10, 28, 45, 62, 80 °C/min).

**Figure 5:**
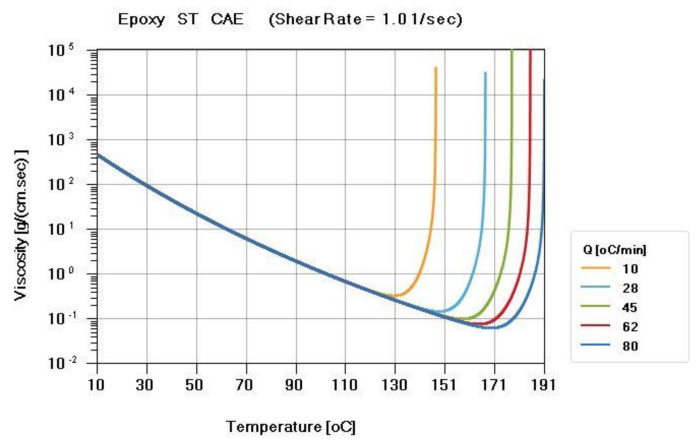
Viscosity curve of the ST resin. The viscosity is measured by a parallel-plate rheometer at different temperatures ramping rates (Q = 10, 28, 45, 62, 80 °C/min) and the viscosity changes with time.

ST resin, which is a thermosetting material, was used in this study. This material was provided by the Metal Industries Research and Development Center, and the material properties were measured by CoreTech System Co., Ltd. A thermal differential scanning calorimeter (DSC) was used to measure the curing kinetics of thermosetting epoxy (Perkin Elmer DSC-8500) (Figure 4). A parallel-plate rheometer (Anton Paar MCR502) was used to measure the viscosity of the epoxy (Figure 5).

Since the resin will solidify as the temperature rises, the curing kinetics must be considered in this study. The curing kinetics model used the Kamal’s model, while the model equation is shown in Section 3.1.

The viscosity of a thermosetting material will decrease with the increase of temperature. Because the viscosity changes with the temperature, the viscosity curve is very important in the research. The viscosity model used the Cross Castro–Macosko model, while the model equation is shown in Section 3.1.

## 3. Simulation

### 3.1. Theoretical Background

For the flow simulations, the principle of conservation of mass, momentum, and energy was used for the governing equation.

- Continuity Equation

The mass conservation of resin during cavity filling is described by a continuity equation:

(1) $$\frac{\partial \rho }{\partial t}+\nabla \cdot \left(\rho \vec{V}\right)=0$$

- Momentum Equation

During the resin filling process, the fluid force changes are described by the momentum equation:

(2) $$\rho \left(\frac{\partial \vec{V}}{\partial t}+\vec{V}\cdot \nabla \vec{V}\right)=-\nabla P+\nabla \cdot \vec{\vec{\tau }}+\rho \vec{g}$$

- Energy Equation

The energy equation is used to describe the conservation of energy in the curing process during the filling process:

(3) $$\rho C_{P}\left(\frac{\partial T}{\partial t}+\vec{V}\cdot \nabla T\right)=\nabla \cdot \left(k\nabla T\right)+\eta {\dot{\gamma }}^{2}+\frac{d\alpha }{dt}\Delta H$$

where t is the time; ρ is the density; $\vec{V}$ is the velocity vector; P is the pressure; $\vec{\vec{\tau }}$ is the stress tensor; $\vec{g}$ is the gravity; $C_{P}$ is the specific heat; T is the temperature; $\dot{\gamma }$ is the shear rate; α is the degree of cure; ΔH is the reaction heat; and η is the viscosity taken as the generalized Cross Castro–Macosko viscosity model [14]:

(4) $$\eta =\frac{\eta_{0}{\left(\frac{\alpha_{g}}{\alpha_{g}-\alpha }\right)}^{C_{1}+C_{2}\alpha }}{1+{\left(\frac{\eta_{0}\dot{\gamma }}{\tau^{*}}\right)}^{1-n^{*}}}$$

(5) $$\eta_{0}=A_{1}\cdot \exp\left(\frac{T_{b}}{T}\right)$$

(6) $$T_{b}=\frac{E}{R}$$

where $\tau^{*}$ is the critical shear stress; $\dot{\gamma }$ is the shear rate; $n^{*}$ is a power law index; *T* is the static temperature; α is the degree of cure; $\alpha_{g}$ is the degree of cure at gel point; $C_{1}$, $C_{2}$ are the fitting constant; $A_{1}$ is the exponential-fitted constant; and $T_{b}$ is the reaction activation energy constant. The values of the Cross Castro–Macosko model constants Equation (4) are given in Table 1.

$\frac{d\alpha }{dt}$ is the curing kinetics taken as Kamal’s model [15]:

(7) $$\frac{d\alpha }{dt}=\left(K_{a}+K_{b}\cdot \alpha^{m}\right)\cdot {\left(1-\alpha \right)}^{n}$$

(8) $$K_{a}=A\cdot \exp\left(\frac{-T_{a}}{T}\right)$$

(9) $$K_{b}=B\cdot \exp\left(\frac{-T_{b}}{T}\right)$$

where $\frac{d\alpha }{dt}$ is cure reaction rate; α is degree of cure; m, n are material constants; $K_{a}$, $K_{b}$ are cure reaction velocity constants; A, B are cure reaction constants; $T_{a}$, $T_{b}$ are activation energies. The values of Kamal’s model constants Equation (7) are given in Table 2.

- Volume of Fluid, VOF.

The free liquid surface problem is quite difficult for numerical calculations. The main reason for this issue is that the boundary of a free liquid surface is a moving boundary, and the boundary changes with time. The free liquid surface must meet the free surface kinematic boundary condition (FSKBC) and the free surface dynamic boundary condition (FSDBC) [16].

The volume of fluid (VOF) [17] is a numerical calculation method that is used to establish the interface boundary conditions for a free surface or two-fluid interfaces. The VOF method is based on defining a fractional volume function that allows a single element to be full, partially filled, or to remain empty. Through the fractional volume function, three areas can be defined in the element as follows:

(10) $$f=\left\{\begin{array}{l} 0,\;\mathrm{the}\;\mathrm{element}\;\mathrm{is}\;\mathrm{empty}.\\ 1,\;\mathrm{the}\;\mathrm{element}\;\mathrm{is}\;\mathrm{full}.\\ 0<f<1,\;\mathrm{there}\;\mathrm{is}\;a\;\mathrm{fluid}\;\mathrm{interface}\;\mathrm{in}\;\mathrm{element}. \end{array}\right.$$

The fractional volume function is governed by a transport equation:

(11) $$\frac{\partial f}{\partial t}+\vec{V}\cdot \nabla f=0$$

The following simple serial averages are adopted in this work to approximate the density and viscosity at the interface between fluid 1 and fluid 2.

(12) $$\rho_{f}=f\cdot \rho_{1}+\left(1-f\right)\cdot \rho_{2}$$

(13) $$\eta_{f}=f\cdot_{1}+\left(1-f\right)\cdot_{2}$$

### 3.2. Simulation Process

**Figure 6:**
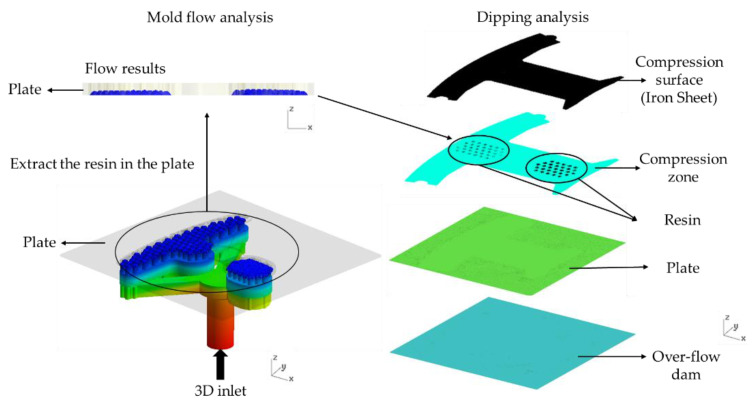
The simulation flow chart.

**Figure 7:**
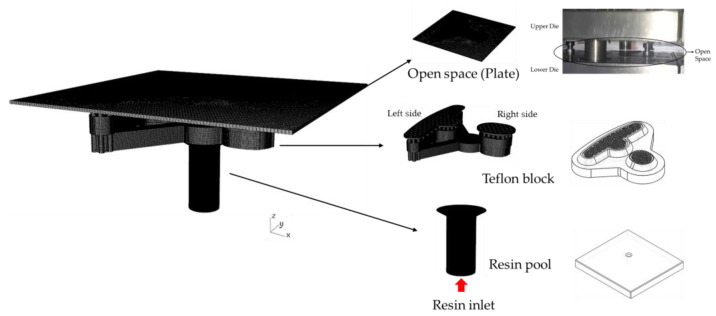
Mesh model used in the filling process.

This study explores the gluing technology used in the iron sheet stacking process of motor cores. The resin is transferred through resin inlet to resin pool by a pump as shown in Figure 6 and Figure 7, so the injection molding process simulation tool, Moldex3D, is used for the filling process simulation. The resin flows through the viscous flow channels and the viscous flow channels are constituted of the resin pool and the Teflon block. When the resin flows through 122 holes in a plate, hemi-spheres of resin will be formed. An iron sheet is then dipped on the emerged resin to form a thin layer of glue to bond iron layers to make a motor core.

During the dipping process, the iron sheet moves with the upper die. Therefore, compression molding process simulation is used to model the dipping process. The emerged hemi-sphere shaped resin in the plate is numerically extracted as the initial condition for the dipping analysis to predict whether the resin is evenly distributed on the iron sheet.

The stacking process is fully elucidated through the use of a mold flow analysis and a dipping analysis in order to establish the simulation approach used in this study.

### 3.3. Mesh Generation

For the purposes of the simulation, the process is divided into two parts, where the mesh is divided into two parts and constructed as a filling process and a dipping process. Rhinoceros 5 was the mesh modeling software used in this study.

#### 3.3.1. Filling Process

**Figure A1:**
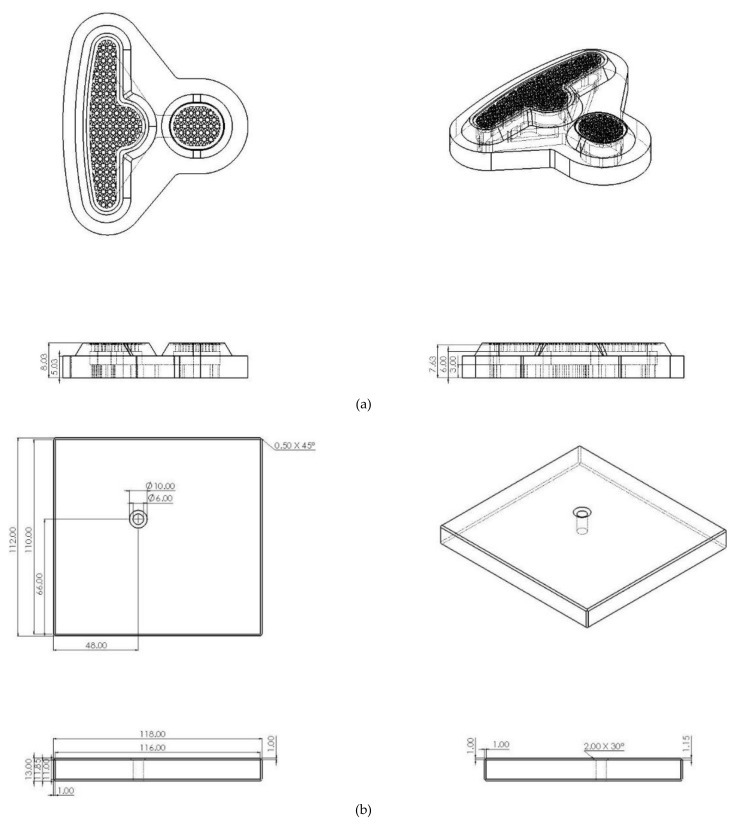
The detail dimensions of the filling model. (Unit: mm) (**a**) The Teflon block; (**b**) The resin pool.

A solid 3D mesh model is created through geometry, as shown in Figure 7. The resin pool and the Teflon block are constructed according to the geometry provided by the Metal Industries Research and Development Center, Kaohsiung, Taiwan. The diameter of the resin inlet is 6 mm, then the resin is divided to the left and right side by three runners (Figure 3b), and finally the resin flows out through 122 holes with 1 mm diameter. The location of the holes is shown in Figure 3a. The detail dimensions of the filling model are shown in Appendix A, Figure A1. The plate is used to simulate the open space of the resin flow out of the system. There are 3.5 million mesh elements, for which the quality is 0.97 based on the skewness.

#### 3.3.2. Dipping Process

**Figure 8:**
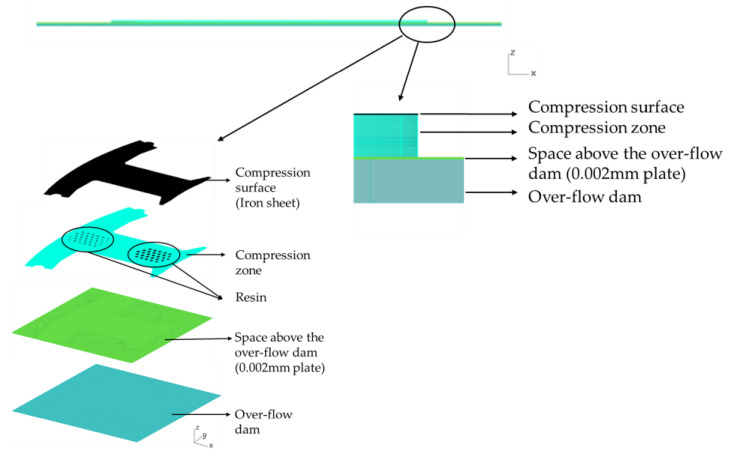
Mesh model used in the dipping process.

The mesh used for the dipping process is composed of a compression surface (iron sheet), a compression zone, a space above the over-flow dam (plate), and the over-flow dam, as shown in Figure 8. The compression zone part simulates the process of gluing the iron sheet. In addition, since the resin on the iron sheet cannot exceed 0.002 mm, the resin is compressed into a space with a thickness of 0.002 mm (the space above the over-flow dam) for the simulation. There is a total of 2.5 million mesh elements, for which the quality is 0.99 based on the skewness.

### 3.4. Process Parameters

The stamping process is used for gluing the motor core. Due to the design of the runner and the module, the flow of the resin is very important to the overall process. Therefore, the flow is predicted through a simulation, and the experimental parameters are input into the mold flow analysis software to simulate and predict the resin flow and gluing results.

#### 3.4.1. Filling Process

In the experiment, the resin was pushed by a pump, for which the pressure was 0.8 MPa, so the injection pressure was set at 0.8 MPa. The mold temperature and the resin temperature were both set to 45 °C based on the data provided by the Metal Industry Research and Development Center, which indicates that the temperature of the resin before entering the module is about 45 °C. The mold flow analysis mold is set at 45 °C because, regardless of whether it passes through the runner in the resin pool or the runner or microstructure in the Teflon block, the resin temperature is still about 45 °C, so the resin is flowing isothermally in the glue module. In the setting of process parameters, the initial conversion rate of plastics is very important. In this study, Moldex3D mold flow analysis software was used to calculate the initial conversion rate. The resin was taken out at room temperature (25 °C) for one day, and the initial conversion rate barely changed. Therefore, the initial conversion rate was set to 0%. The process parameters can be shown in Table 3. In the experiment, the volume flow rate is 0.00053 cm^3^/s. The volume flow rate cannot be set in the simulation analysis, so it can only be measured based on the filling time. Since the total volume of the mesh model established in this paper was 4.16 cm^3^, the filling time was set to 416 s, for which the volume flow rate was 0.01 cm^3^/s. The reason why the volume flow rate of this study was set at 0.01 cm^3^/s is explained in the Results and Discussion section of this paper.

#### 3.4.2. Dipping Process

The parameters for the dipping process are shown in Table 4. The compression speed and compression force, which is the stamping speed and stamping force in the stamping process, respectively, were set based on the Metal Industries Research and Development Center guidelines. The thickness of the compression zone was 0.5 mm because the thickness of the compression zone only addressed the area where the iron sheet is prepared to glue the resin. The compression time was obtained by the compression gap divided by the compression speed. The resin and mold temperature settings were also set based on the data provided by the Metal Industries Research and Development Center. The initial conversion rate setting was calculated using Moldex3D software. From the filling process to the gluing stage, the resin in the module does not flow over 1 hour, so the conversion rate remained at 0%.

### 3.5. Mold Flow Analysis Software

A commercially available CAE software Molde×3D 2020 R1 special edition for free surface simulation was used for the analysis in this study. It is a CAE software used mainly for injection molding and compression molding simulations.

## 4. Results and Discussion

This section discusses the influence of gravity and the flow rate on the simulation and also discusses the resin distribution during the dipping process and the distribution of the air traps, after which a comparison of the results with the experimental results are discussed.

### 4.1. The Influence of Gravity on Simulation

**Figure 9:**
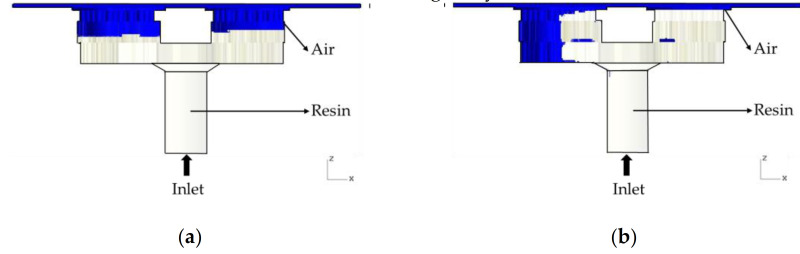
The effect of gravity on simulation (**a**) considering gravity and (**b**) not considering gravity.

The effect of gravity in this study was very significant. Figure 9 shows the influence of gravity on the simulation. If gravity is ignored, there is a slightly unreasonable flow situation, as shown in Figure 9. When gravity is taken into account, the results are close to actual conditions. Therefore, gravity must be taken into consideration.

### 4.2. The Influence of Flow Rate on Simulation

**Figure 10:**
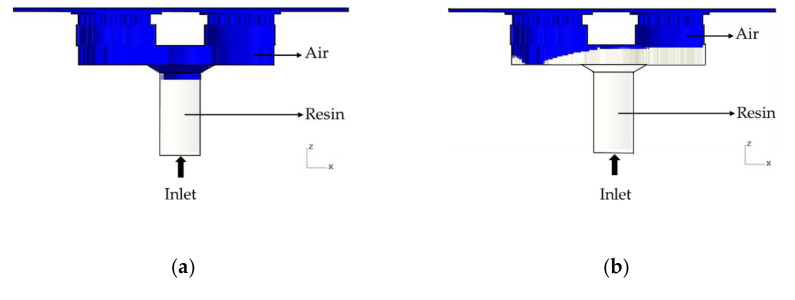

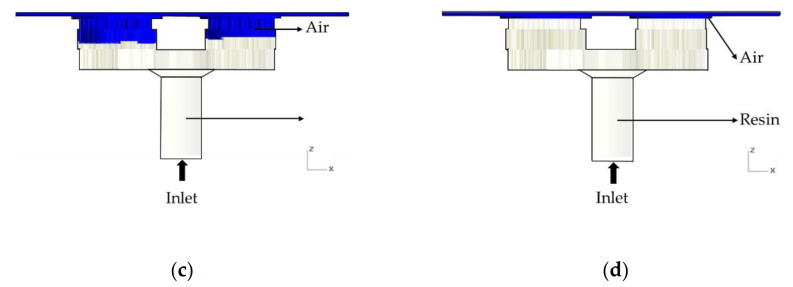
The flow rate is 0.01 cm^3^/s. (**a**) Filling 10%; (**b**) Filling 30%; (**c**) Filling 50%; (**d**) Filling 70%.

**Figure B1:**
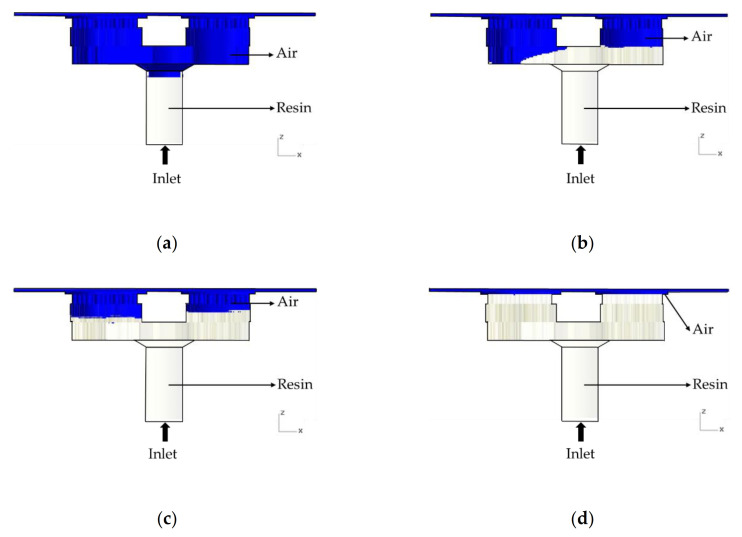
The flow rate is 0.02 cm^3^/s. (**a**) Filling 10%; (**b**) Filling 30%; (**c**) Filling 50%; (**d**) Filling 70%.

**Figure B2:**
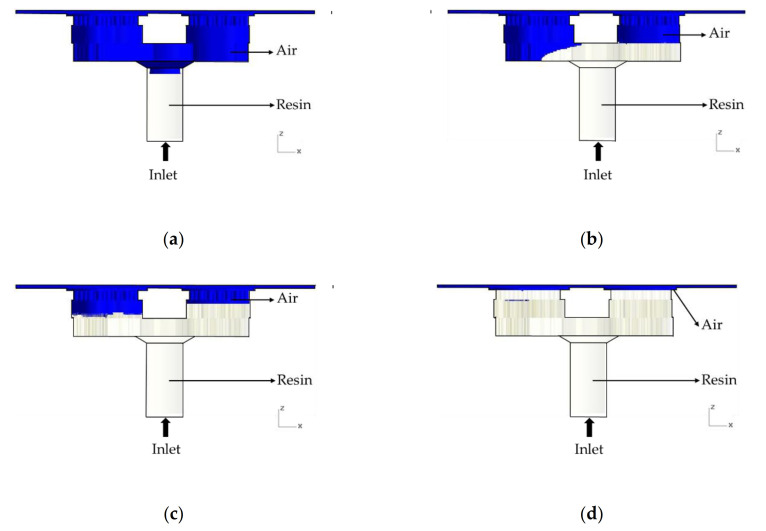
The flow rate is 0.03 cm^3^/s. (**a**) Filling 10%; (**b**) Filling 30%; (**c**) Filling 50%; (**d**) Filling 70%.

**Figure B3:**
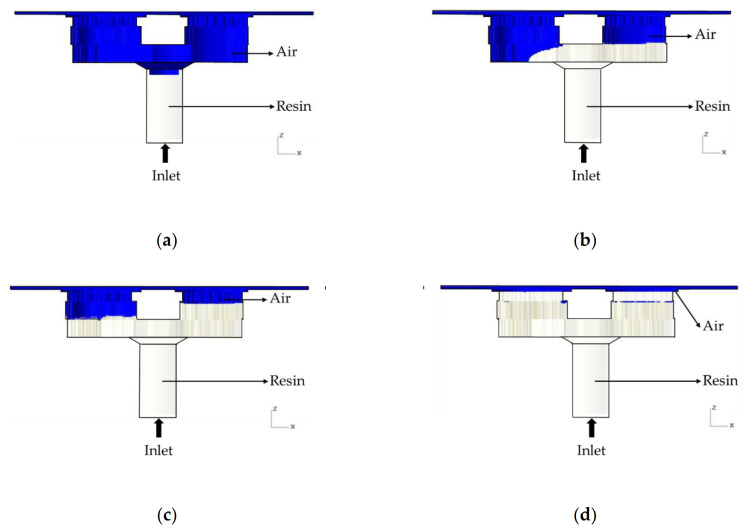
The flow rate is 0.04 cm^3^/s. (**a**) Filling 10%; (**b**) Filling 30%; (**c**) Filling 50%; (**d**) Filling 70%.

**Figure B4:**
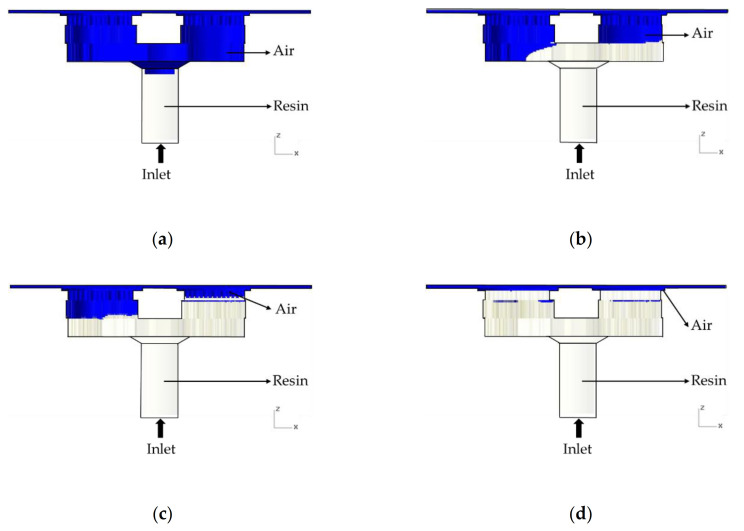
The flow rate is 0.05 cm^3^/s. (**a**) Filling 10%; (**b**) Filling 30%; (**c**) Filling 50%; (**d**) Filling 70%.

In this simulation, if the flow rate is set to be the same as the experiment in the mold flow analysis, the air resistance will be too large, which will cause the resin to fail to flow smoothly and cause the simulation to stop. Therefore, different fill times were set, which made the flow rate respectively 0.01 (see Figure 10), 0.02, 0.03, 0.04, and 0.05 cm^3^/s. The simulation results of the flow rate 0.02, 0.03, 0.04, 0.05 cm^3^/s are shown in Appendix B, Figure B1, Figure B2, Figure B3 and Figure B4. From the simulation results of different flow rates, it can be seen that the flow trend is similar at different flow rates. In this study, the flow rate closest to the experimental conditions was used for the simulation (0.01 cm^3^/s). Experiment and simulation results of the flow trend in the plate are shown in Table 5.

### 4.3. Results for Dipping an Iron Sheet

**Figure 11:**
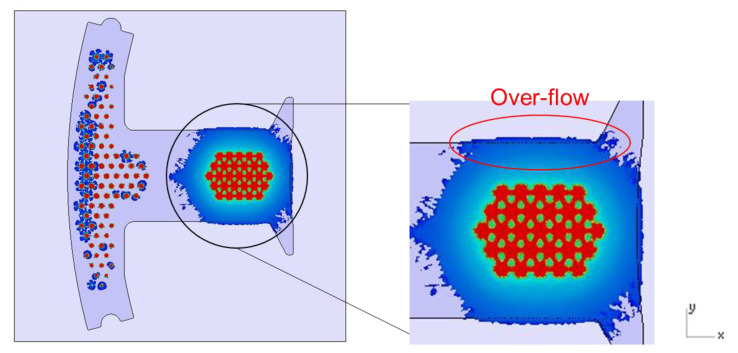
Top view of the dipping process results.

**Figure 12:**
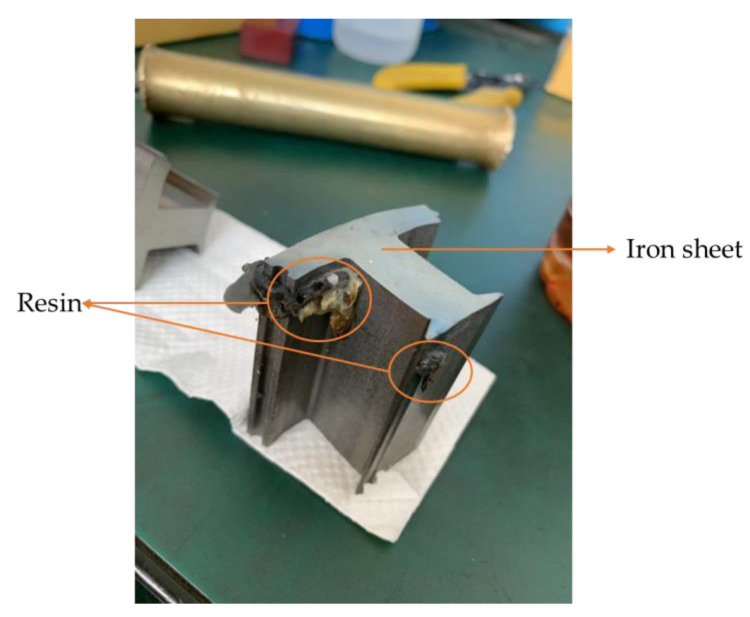
Schematic diagram of bumps caused by resin overflow.

From the results of the filling process (Table 5), it can be seen that the resin emerges first from the right side, then the middle of the left side, and finally from the two sides of the left T-shape. There is obviously more resin on the right side than that on the left side. The emerged resin in the plate is numerically extracted, and then the resin is used as the initial condition for the dipping analysis. The simulation result of the dipping process is shown in Figure 11. The distribution of resin is uneven, which will reduce the adhesion quality of the iron sheets, and there is an over-flow on the right side, which will cause bumps when the iron chips are stacked on each other (the resin area exceeds the size of the iron sheet) (see Figure 12).

### 4.4. Air Traps Results 

**Figure 13:**
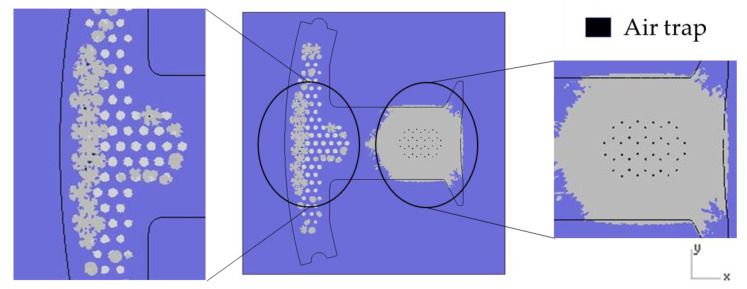
Position of air traps.

The simulation was very important not only in terms of predicting the flow of resin during the dipping process but also in terms of predicting the occurrence of air traps. The occurrence of air traps may reduce the adhesive area. In addition to insufficient adhesion, the subsequent heating process for the iron sheet may also produce a popcorn effect, which may decrease product quality. From Figure 13, it can be seen that most of the air traps are located on the right half. This phenomenon is due to too much resin on the right half, which causes the gas to not discharge smoothly during the dipping process.

### 4.5. Comparison of the Target Value and the Simulation Results

Since it is not easy to measure the resin volume in the experiment, the resin volume can be obtained by the estimated resin area (320.8 mm^2^) times the resin thickness (0.002 mm) is 0.64 cm^3^, which can be used as a comparison basis for simulation.

**Figure 14:**
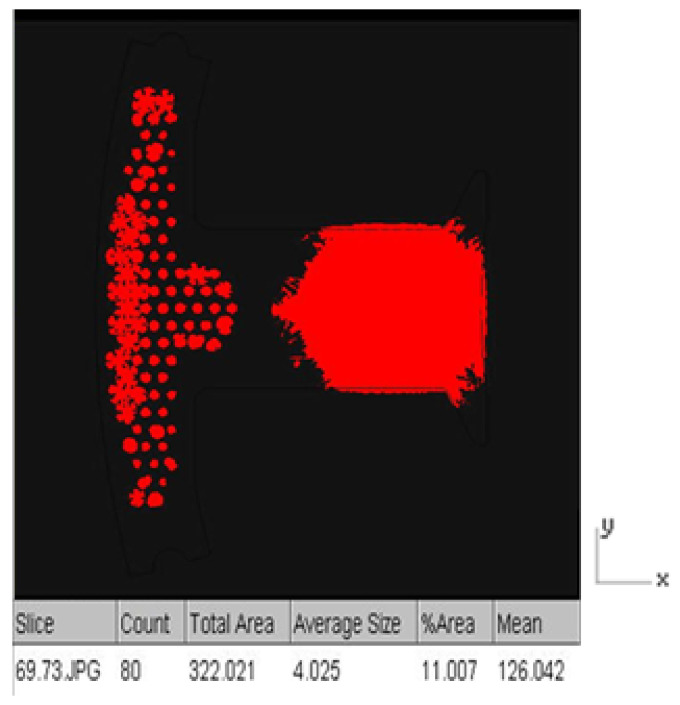
ImageJ measurement results.

Image analysis software (ImageJ) was used to analyze the resin area associated with the dipping results, which was 322.021 mm^2^ (Figure 14), and the volume was 0.644 mm^3^, resulting in a simulation error of 0.6%.

## 5. Conclusions

This article was focused on the gluing technology used in the stamping process. The gluing method was relatively similar to other joining methods (welding, riveting), but it had advantages including lowering iron loss, smoothing the magnetic circuit, and obtaining higher overall rigidity. In this study, Moldex3D mold flow analysis software was used to predict the flow of the resin due to the design of the module and runners and the process used to stick the iron sheet during the motor core die-bonding process, in order to observe the resin flow. In this research, the flow of the plastic in the module was simulated first, and then the flow results were taken as the condition before dipping in order to predict the plastic dipping process under specific conditions.

Based on the research results, the following conclusions could be drawn:

1. The properties of the fluid in the mold flow analysis are very important. In the present research, Kamal’s reaction dynamic model and the Cross Castro–Macosko viscosity model were used to define the material parameters, so the mold flow analysis could better describe the material characteristics.
2. A mold flow analysis and a dipping analysis were used to compare the entire motor core filling and dipping process. The prediction results were very good for both the flow in the filling process and the dipping process, and it was possible to effectively predict the area where the air traps would occur when glued based on the results of the analytical software.
3. Image analysis software (ImageJ) was used to analyze the resin area in the simulation results, for which the resulting area was 322.021 mm^2^ and the volume was 0.644 mm^3^. The target value of area is 320.8 mm^2^ and the volume is 0.64 mm^3^. The error value for the simulation results was 0.6%, which may have been caused by extracting the resin.
4. The runner design will cause the resin to flow unevenly and there will be over-flow during the dipping process.

## Figures and Tables

**Table 1:** Values of the Cross Castro–Macosko model constants. The constants were measured by a parallel-plate rheometer.

Process Parameters	Parameter Values	Unit
$\alpha_{g}$	0.3	-
$C_{1}$	5.3193	-
$C_{2}$	−10	-
$A_{1}$	$1\times 10^{-9}$	g/(cm·s)
$T_{b}$	7996	K
$n^{*}$	0.8	-
$\tau^{*}$	10	dyne/cm^2^

**Table 2:** Values of Kamal’s model constants. The constants were measured by a DSC.

Process Parameters	Parameter Values	Unit
m	0.75648	-
n	2.0614	-
A	228.29	1/s
B	$2.5826\times 10^{7}$	1/s
$T_{a}$	14,586	K
$T_{b}$	8619	K

**Table 3:** The parameters of the filling process.

Process Parameters	Parameter Values
Injection pressure (MPa)	0.8
Melt temperature (°C)	45
Mold temperature (°C)	45
Initial conversion (%)	0
Filling time (s)	416

**Table 4:** The parameters of the dipping process.

Process Parameters	Parameter Values
Compression time (s)	0.273
Compression gap (mm)	0.5
Compression speed (mm/s)	1.83
Compression force (tf)	0.022
Resin temperature (°C)	45
Mold temperature (°C)	45
Initial conversion (%)	0

**Table 5:** Experiment and simulation results of flow trend in the plate. Since the filling process of the experiment is not easy to observe, there is a large gap in the total filling time. Therefore, the time difference is used to determine whether the trend is similar.

Experiment	Simulation	Experiment (Δ*t*)	Simulation (Δ*t*)
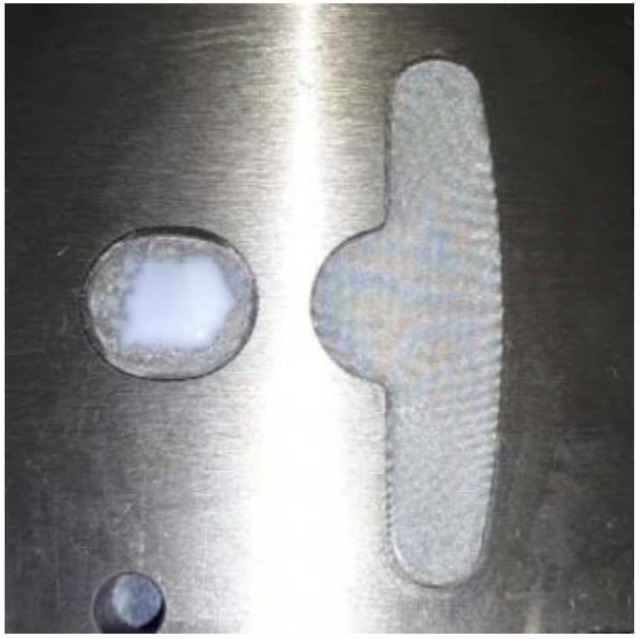 40 s	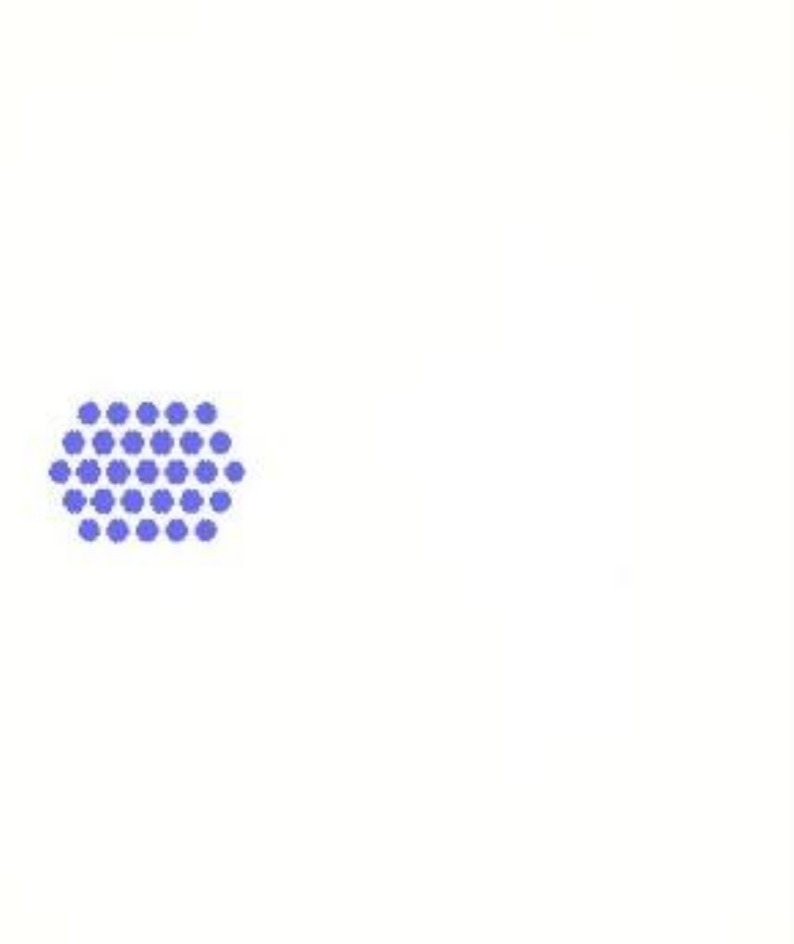 279 s	-	-
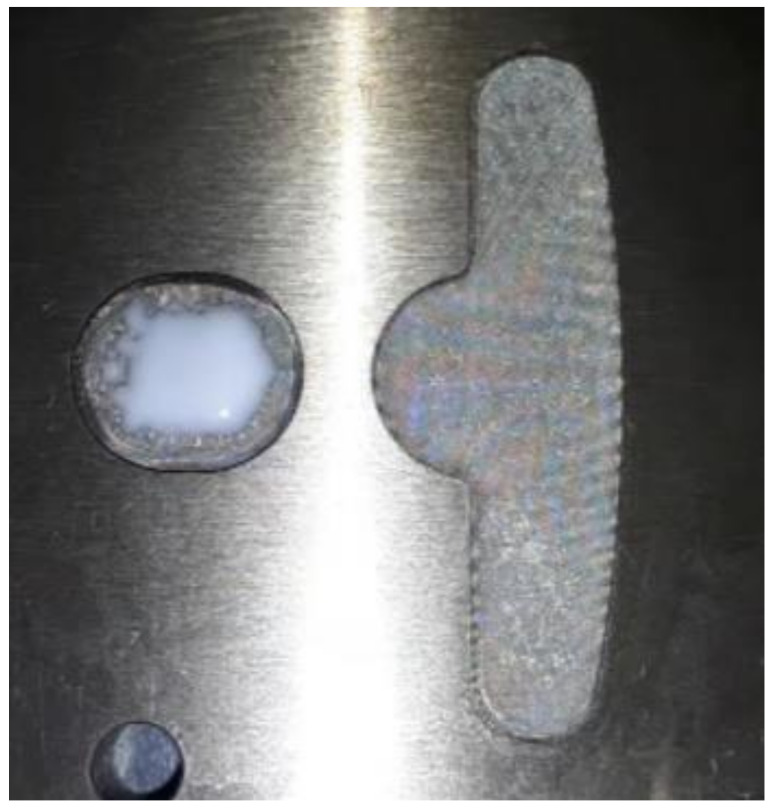 43 s	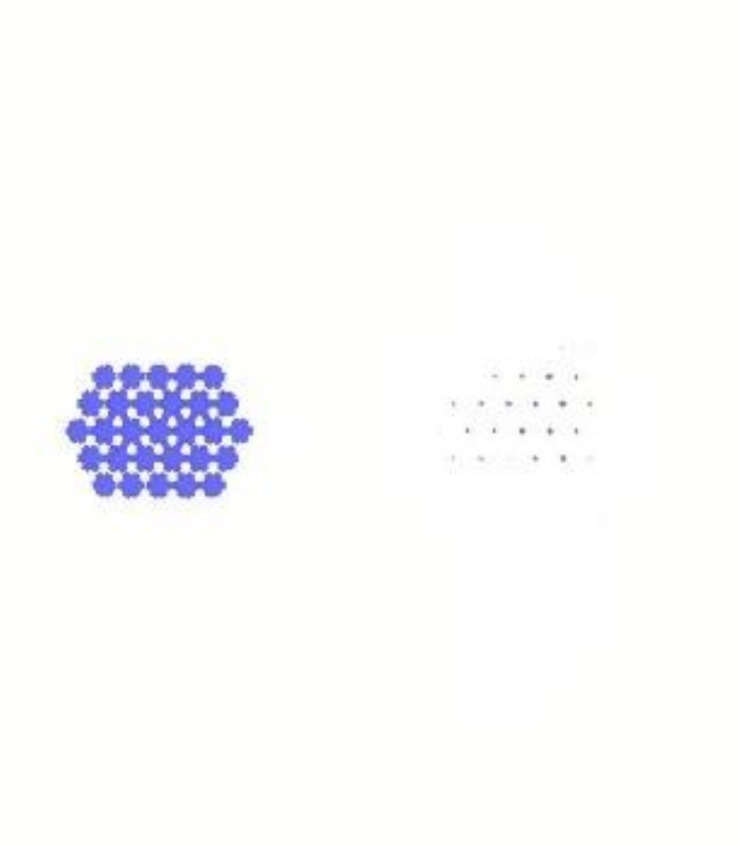 283 s	3 s	4 s
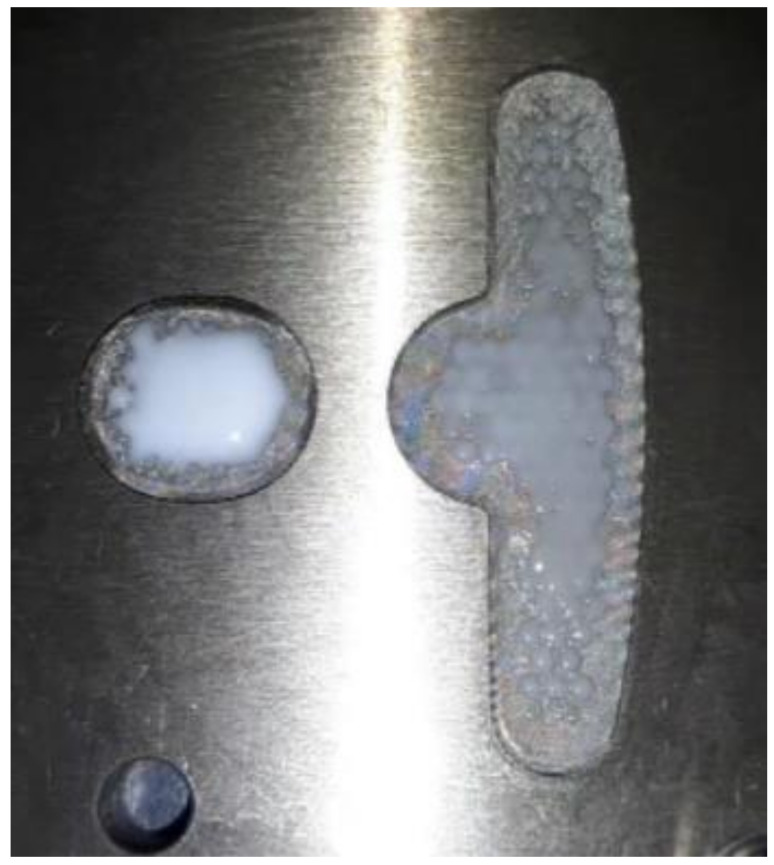 45 s	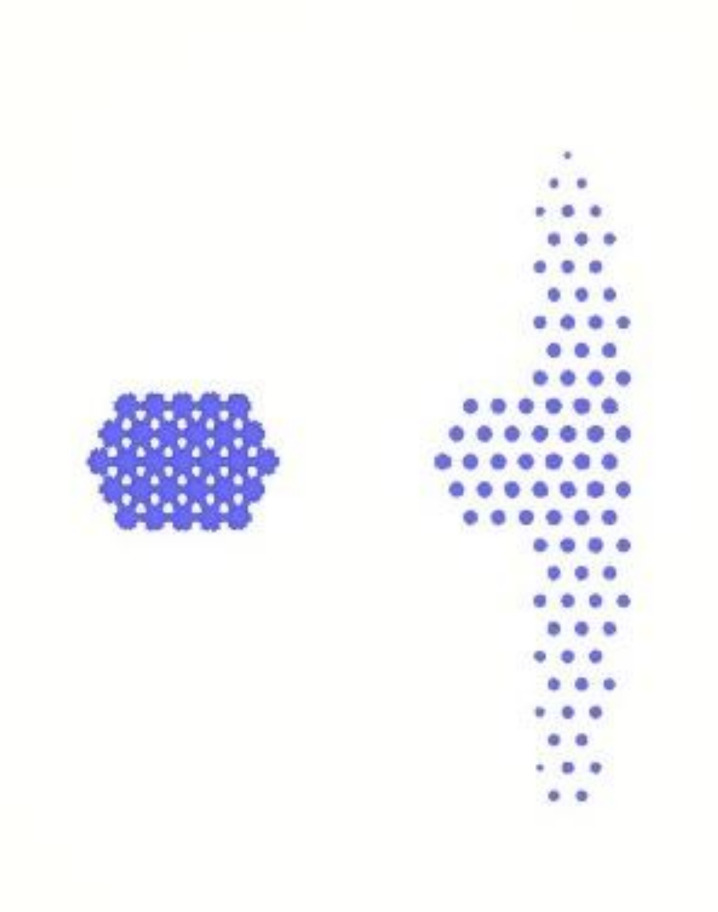 285 s	2 s	2 s
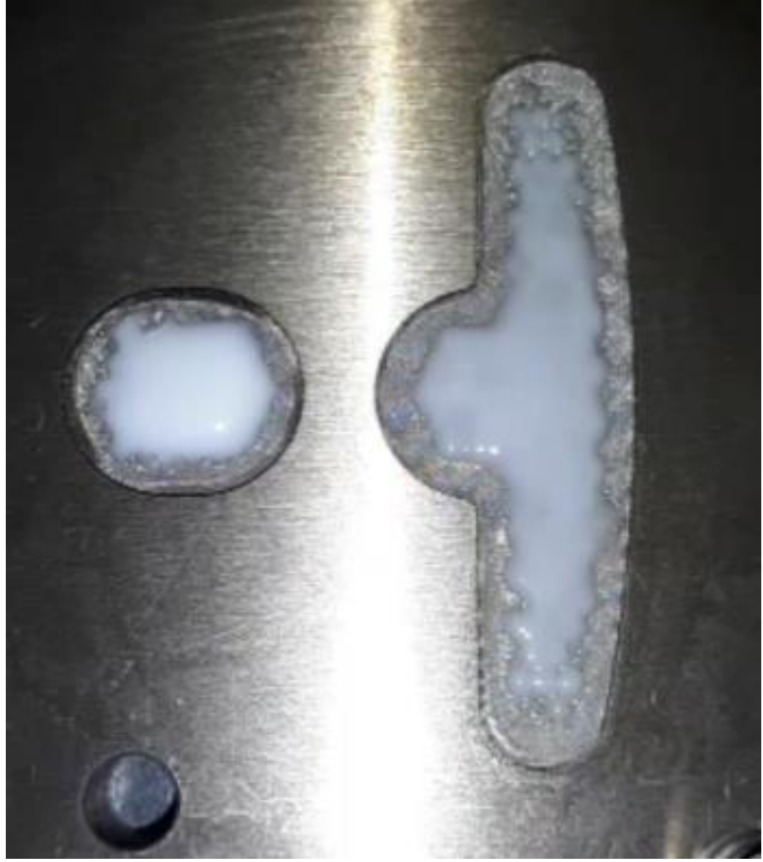 48 s	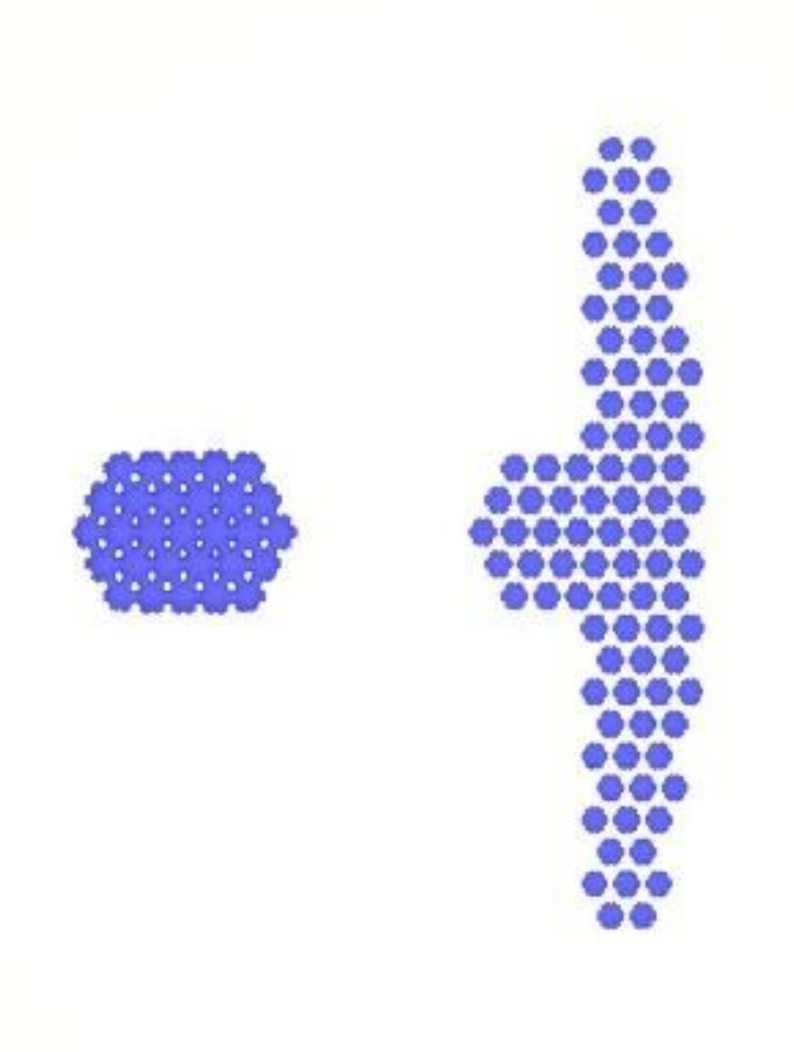 289 s	3 s	4 s

## Data Availability

The data presented in this study are available on request from the corresponding author.
